# Supplementary material for: Inter-domain microbial diversity within the coral holobiont Siderastrea siderea from two depth habitats
Source: PeerJ. 2018 Feb 9;6:e4323. doi: 10.7717/peerj.4323 (PMC5808317; doi:10.7717/peerj.4323)
Supplement: Supplemental Information 3 [file peerj-06-4323-s003.docx]

Treatment of raw Illumina MiSeq data with Mothur

January, 2016

**Below the commands executed in Mothur for the treatment with brief descriptions. All is based on the MiSeq SOP, with slight additions.**

**Assay: 14 samples, cp23S marker, host *S. siderea***

**make.contigs(ffastq= , rfastq= ,oligos= , checkorient=t, pdiffs = 3 , processors=1)** # joining of forward and reverse reads, using the .oligos file, this the following text document, containing barcode and primer sequences ending with ' .oligos' .

barcode GATGAGTC NONE FKSSSYM1

barcode GATGCAAC NONE FKSSSYM2

barcode GATGCACT NONE FKSSSYM3

barcode GATGCATG NONE FKSSSYM4

barcode GATGCCAC NONE FKSSSYM5

barcode GATGCCAG NONE FKSSSYM6

barcode GATGCCCA NONE FKSSSYM7

barcode GATGCGAG NONE FKSSSYM9

barcode GATGCGCG NONE FKSSSYM8

barcode GATGGACG NONE FKSSSYM10

barcode GATGGACT NONE FKSSSYM11

barcode GATGGATC NONE FKSSSYM12

barcode GATGGATG NONE FKSSSYM13

barcode GATGGCAA NONE FKSSSYM14

primer TCAGTACAAATAATATGCTG CGCCCCAATTAAACAGT

**screen.seqs(fasta= , group= , maxambig=0, maxlength=350, maxhomop=5)
unique.seqs(fasta= )
count.seqs(name= , group= )**
**pcr.seqs(fasta=, start=137, end=563, keepdots=F, processors=1) )** # Here, the reference alignment is Cp23S_reference_alignment.fasta. 'Start' and ' end' parameters are the positions of forward and reverse primers in this reference alignment.

**align.seqs(fasta= , reference= )**
**summary.seqs(fasta= , count= )**
**screen.seqs(fasta= , count= , summary= , start=1, end=426, maxhomop=8)**
**filter.seqs(fasta= , vertical=T, trump=.)**
**unique.seqs(fasta= , count= )**
**pre.cluster(fasta= , count= , diffs=1)**
**chimera.uchime(fasta= , count= , dereplicate=t)**
**remove.seqs(fasta= , accnos= )**

**classify.seqs(fasta= , count= , reference= , taxonomy= , cutoff=60)** # All sequences are classified along the reference file (Cp23S_reference_classify.fasta and Cp23S_reference_classify.tax)

**remove.lineage(fasta= , count= , taxonomy= , taxon=unknown)
dist.seqs(fasta= , cutoff=0.20)**

**cluster(column= , count=, method=nearest)**
**make.shared(list= , count= , label=0.03)**
**count.groups(shared= )**

**degap.seqs(fasta= )
split.abund(fasta= , list= , count= , label=0.03, cutoff=10)**

**make.shared(list= , count= , label=0.03)**

**get.oturep(fasta= , count= , list= , sorted=bin, method=abundance)**
**classify.seqs(fasta= , count= , template= , taxonomy= , cutoff=80)**

**classify.otu(list= , count= , taxonomy= , cutoff=80)**

The two files made from **make.shared()** and **classify.otu()** are combined in R into the OTU table.

**get.mimarkspackage(oligos= )** # Preparing for submission to SRA

**make.sra(file= , oligos= , project= , mimark= , pdiffs = 3 , checkorient=t)**

**Assay: 14 samples, 16S marker, host *S. siderea***

**make.contigs(ffastq= , rfastq= ,oligos= , checkorient=t, pdiffs=3, processors=1)** # joining of forward and reverse reads, using the .oligos file, this the following text document, containing barcode and primer sequences ending with ' .oligos' .

barcode CGTAGATA NONE FKSSBAC1

barcode CGTAGGCT NONE FKSSBAC2

barcode CGTATTCA NONE FKSSBAC3

barcode CGTATTTC NONE FKSSBAC4

barcode CGTCAAGA NONE FKSSBAC5

barcode CGTCACAG NONE FKSSBAC6

barcode CGTCCAGG NONE FKSSBAC7

barcode CGTCGCAT NONE FKSSBAC9

barcode CGTCTGAA NONE FKSSBAC8

barcode CGTGAGAC NONE FKSSBAC10

barcode CGTGATAA NONE FKSSBAC11

barcode CGTGGGAC NONE FKSSBAC12

barcode CGTGGTCA NONE FKSSBAC13

barcode CGTTCACG NONE FKSSBAC14

primer AGRGTTTGATCMTGGCTCAG TACNGCGGCKGCTG

**screen.seqs(fasta= , group= , maxambig=0, minlength=380, maxlength=520, maxhomop=8)
unique.seqs(fasta= )**
**count.seqs(name= , group= )**
**pcr.seqs(fasta=, start=1, end=13128, keepdots=F, processors=1)** # Here, the reference alignment (the SILVA v123 alignment is used.

**align.seqs(fasta= , reference= )**

**screen.seqs(fasta= , count= , summary= , start=1043, end=13126, maxhomop=8)**
**filter.seqs(fasta= , vertical=T, trump=.)**
**unique.seqs(fasta= , count= )**
**pre.cluster(fasta= , count= , diffs=3)**
**chimera.uchime(fasta= , count= , dereplicate=t, minh = 0.1, xn = 4)**
**remove.seqs(fasta= , accnos= )**
**classify.seqs(fasta= , count= , reference= , taxonomy= , cutoff=60)** # using the SILVA v123 reference files

**remove.lineage(fasta= , count= , taxonomy= , taxon=Mitochondria;-Chloroplast;, alignreport= )** # Sequences that were not identified are removed. It is also possible to remove specific taxa. Before using the SILVA reference alignment, this and the previous step were also performed with the GreenGenes alignment

**dist.seqs(fasta= , cutoff=0.30)**

**cluster(column= , count=, method=nearest)
make.shared(list= , count= , label=0.03)**
**count.groups(shared= )**
**degap.seqs(fasta= )**
**split.abund(fasta= , list= , count= , label=0.03, cutoff=10)**
**make.shared(list= , count= , label=0.03)**
**get.oturep(fasta= , count= , list= , sorted=bin, method=abundance)**
**classify.seqs(fasta= , count= , template= , taxonomy= , cutoff=80)**
**classify.otu(list= , count= , taxonomy= , cutoff=80)**

The two files made from **make.shared()** and **classify.otu()** are combined in R into the OTU table.

**get.mimarkspackage(oligos= )**
**make.sra(file= , oligos= , project= , mimark= , pdiffs=3, checkorient=t)**

**Assay: 14 samples, ITS2 marker, host *S. siderea***

**make.contigs(ffastq= , rfastq= ,oligos= , checkorient=t, pdiffs=2 processors=1)** # joining of forward and reverse reads, using the .oligos file, this the following text document, containing barcode and primer sequences ending with ' .oligos' .

barcode TCTCTGGA NONE FKSSFUN1

barcode TCTCTTCA NONE FKSSFUN2

barcode TCTCTTCG NONE FKSSFUN3

barcode TCTGACCA NONE FKSSFUN4

barcode TCTGAGCA NONE FKSSFUN5

barcode TCTGAGGA NONE FKSSFUN6

barcode TCTGAGGT NONE FKSSFUN7

barcode TCTGAGTT NONE FKSSFUN9

barcode TCTGCAAT NONE FKSSFUN8

barcode TCTGCACT NONE FKSSFUN10

barcode TCTGCAGT NONE FKSSFUN11

barcode TCTGCATA NONE FKSSFUN12

barcode TCTGCCAT NONE FKSSFUN13

barcode TCTGCCCT NONE FKSSFUN14

primer GCATCGATGAAGAACGCAG CCGCTTATTGATATGC

**screen.seqs(fasta= , group= , maxambig=0, minlength=275, maxlength=500, maxhomop=8)**
**unique.seqs(fasta= )**
**count.seqs(name= , group= )**
**pre.cluster(fasta= , count= , diffs=4)**
**chimera.uchime(fasta=, count=, dereplicate=t, reference=self)**
**remove.seqs(fasta= , accnos= )**
**classify.seqs(fasta= , count= , template= , taxonomy= , cutoff=50, processors=1)**
**remove.lineage(fasta= , count= , taxonomy= , taxon= Animalia-unknown)**
**pairwise.seqs(fasta=, cutoff=0.45, processors=1)**
**cluster(column= , count= , method = nearest)**
**make.shared(list= , count= , label=0.05)**
**split.abund(fasta= , list= , count= , label=0.03, cutoff=10)**
**make.shared(list= , count= , label=0.03)**
**get.oturep(fasta= , count= , list= , sorted=bin, method=abundance)**
**classify.seqs(fasta= , count= , template= , taxonomy= , cutoff=60, processors=1)**
**classify.otu(list= , count= , taxonomy= , cutoff=60 , processors=1)**

The two files made from **make.shared()** and **classify.otu()** are combined in R into the OTU table.

**get.mimarkspackage(oligos= )**
**make.sra(file= , oligos= , project= , mimark= , pdiffs=2, checkorient=t)**
